# Dietary supplementation with *Bacillus velezensis* and *Pichia guilliermondii* improves growth performance through intestinal morphology and functionality enhancement in weaning piglets

**DOI:** 10.1371/journal.pone.0332920

**Published:** 2025-12-04

**Authors:** Marcella Massimini, Gianmarco Del Vecchio, Francesca Ciaramellano, Isa Fusaro, Amilcare Barca, Mariarita Romanucci, Melania Giammarco, Chiara Amatetti, Benedetta Bachetti, Marco Di Domenico, Gianluca Nicolai, Barbara Secondini, Andrea Di Credico, Vittoria Di Tecco, Angela Di Baldassarre, Cesare Cammà, Tiziano Verri, Sergio Oddi, Leonardo Della Salda

**Affiliations:** 1 Department of Veterinary Medicine, University of Teramo, Teramo, Italy; 2 Department of Biological and Environmental Sciences and Technologies, University of Salento, Lecce, Italy; 3 European Center for Brain Research/Institute for Research and Health Care (IRCCS) Santa Lucia Foundation, Rome, Italy; 4 Department of Experimental Medicine, University of Salento, Lecce, Italy; 5 R&D Division, C.I.A.M. Srl, Ascoli Piceno, Italy; 6 Istituto Zooprofilattico Sperimentale dell’Abruzzo e del Molise, Teramo, Italy; 7 Reprogramming and Cell Differentiation Lab, Center for Advanced Studies and Technology (CAST), Chieti, Italy; University of Life Sciences in Lublin, POLAND

## Abstract

The post-weaning period in piglets is often associated with significant changes in intestinal structure and function, which can negatively affect growth and performance. This study aimed to evaluate the effects of dietary supplementations with prebiotics and/or probiotics on intestinal morphology and functionality during the post-weaning phase. Piglets were supplemented with different diets, including combinations of *Pichia guilliermondii* (prebiotic, PRE) and *Bacillus velezensis* (probiotic, PRO), and their growth performance, intestinal histology, and functionality were assessed. Growth performance was evaluated in the post-weaning period, with a notable improvement in weight gain observed in piglets receiving the combined prebiotic/probiotic supplementation. Morphometric analysis revealed significant differences in intestinal morphology, particularly in the small intestine. The PRE/PRO combination was most effective in improving villus size in the duodenum and ileum at early post-weaning stages, while the jejunum showed a greater sensitivity to weaning stress. Molecular analysis indicated diet-dependent changes in the expression of key markers involved in nutrient absorption and mucosal integrity, including *SLC15A1* and *TJP1*. Additionally, 16S rRNA sequencing revealed diet-related changes in gut microbiota composition where α-diversity and β-diversity increased independently of the diets and, specific beneficial taxa (e.g., Lachnospiraceae) were enriched in the supplemented groups. These microbial shifts were consistent with the improved mucosal thickness observed in the colon, suggesting a potential contribution of microbial modulation to epithelial integrity of the large intestine. Overall, the results suggest that the synbiotic supplementation of prebiotics and probiotics can enhance intestinal integrity in both the small and large intestine, promote growth, and improve overall gut health in post-weaning piglets.

## 1. Introduction

In intensive farming systems, piglets are typically weaned at 3–4 weeks of age, significantly earlier than the natural weaning period of 10–17 weeks [1,2]. This practice aims to reduce the inter-birth interval in sows, thereby optimizing farm profitability. However, early weaning of piglets represents one of the most stressful events in their lifecycle, since this transition requires rapid adaptation to significant changes in both physical and social environments of animals having an immature immune system [3] and a low digestive capacity [4]. In fact, weaning often results in a severe growth check and diarrhoea with anorexia and undernutrition [3], due to the instability of the intestinal microbiota [5] and the subsequent predisposition of piglets to opportunistic pathogen infections [6,7]. The combination of these phenomena is called post-weaning syndrome (PWS) (for a review see, e.g., [8,9]).

Probiotics and prebiotics are well-established nutritional strategies for improving gut health in piglets and avoiding the use of antibiotics [10]. Probiotics consist of live microorganisms conferring health benefits to the host when administered in sufficient quantities [11,12]. *Bacillus* spp. are among the most commonly used microorganisms in probiotic products. *Bacillus* is a genus of Gram-positive rod-shaped bacteria forming endospores, which make them suitable for high-temperature industrial processes such as pelletizing and extrusion, also conferring greater survivability at the gastric acidic pH [13]. Several studies have shown that various strains of *Bacillus* may improve animal growth performance by enhancing digestibility and nutrient absorption, while reducing the incidence of intestinal pathologies [14]. Furthermore, many *Bacillus* strains produce bacteriocins, proteins capable of directly inhibiting the growth of pathogenic bacteria [15]. One of the most studied and used probiotic species is *B. subtilis*, which is mainly found in soil. The mechanisms of action of *B. subtilis* in the intestine appear to be multifactorial and can vary depending on the specific conditions of the organism [16,17]. Calsporin® is a registered trademark of probiotic dietary supplement based on live spores and authorized by the European Food Safety Authority (EFSA) for use in sows, suckling and weaned piglets, fattening pigs, broiler chickens, laying hens, ornamental fish, and dogs. Initially, Calsporin® was sold as *Bacillus subtilis* strain C-3102A, but in 2021 it was reclassified as *Bacillus velezensis* DSM 15544 [18]. As a recently identified species, *B. velezensis* remains relatively unknown to date, although its mechanisms of action as a probiotic may include the production of bioactive metabolites with antibacterial and antifungal properties [19].

Prebiotics were initially defined as “nondigestible food ingredients that beneficially affect the host by selectively stimulating the growth and/or activity of one or a limited number of bacterial species, and thus attempting to improve host health” [20,21]. CitriStim® is a powder blend obtained from the fermentation of citric acid by *Pichia guilliermondii*, a patented strain of yeast. This supplement product consists of yeast cell walls that are heat-inactivated and subsequently dried after the fermentation process. Generally, the yeast cell wall fraction accounts for approx. 20–30% of the dry weight of the whole cell. This portion is composed of 85–90% polysaccharides and 10–15% crude proteins [22,23]. Specifically, yeast cell walls are a source of mannanoligosaccharides (MOS) and β-glucans. MOS may provide positive effects on intestinal health through their ability to bind pathogens within the intestine, thus preventing their adhesion to the host epithelium [24]. On the other hand, β-glucans may act by binding to membrane receptors of innate immune cells, located in the mucosal lining of the small intestine, which signal the immune system to prepare for potential pathogenic threats [25,26]. In swine husbandry, CitriStim® has been studied as a dietary supplement for gestating and lactating sows, acting as an immunomodulator and demonstrating improvement in their reproductive performance [27,28].

Assessing intestinal morphometry together with biomarkers of gastrointestinal (GI) epithelial functionality (such as mucus production, peptide transport and tight junctions) is crucial for evaluating the well-being status and absorptive capacity of the post-weaning intestine [29,30], as well as for studying the effects of dietary supplementation with pro- and/or prebiotics [31,32]. In fact, the GI tract of weaning piglets undergoes adaptations to the dietary transition from highly digestible milk to a plant-based diet, leading to modifications of villus morphology in the small intestine [6,33,34]. Previous studies suggested a correlation between post-weaning feed intake and villus size, potentially influencing overall nutrient capture and utilization efficiency [4]. Nutrient absorption in the small intestine relies on factors such as length, density, and placement of intestinal villi, as well as the size and density of enterocyte microvilli with a consequent increase of zootechnical performance [35,36]. Furthermore, during PWS, modifications in intestinal mucins and enzyme activities may occur, resulting in reduced performance and capacity for digestion and absorption [6,29,37,38]. As demonstrated in various species including rats, mice, humans, and pigs, the neutral to acidic mucins ratio typically increases from birth to the weaning period and then decreases post-weaning [39]. Further significant biomolecular, biochemical and/or physiological changes include transient increase in mucosal permeability, disruptions in absorptive-secretory electrolyte balance, and shifts in local inflammatory cytokine patterns [40]. These responses usually occur in two distinct temporal patterns: an acute phase immediately following weaning, followed by an adaptive and maturational phase starting at about day 5 [39]. In this framework, probiotics may improve the intestinal absorption of nutrients, such as dietary amino acids and small peptides.

Although the effects of Calsporin® and CitriStim® on pig zootechnical performances and faecal score have been previously investigated, limited data are available on intestinal morphology of pigs fed with *Bacillus subtilis* [19,30,41,42]. As well, the morphological modifications associated with the use of a mixture containing *Pichia guilliermondii* were only recently described in piglets with respect to villi morphometry and faecal score, although there is no information about its effects as a single agent on intestinal morphology and functionality [27,43].

The objective of this study was to evaluate the effects of oral administration of the probiotic *Bacillus velezensis* DSM 15544, the prebiotic *Pichia guilliermondii*, and their combination on growth performance, intestinal morphometric parameters, mucosal turnover, and faecal score, alongside the analysis of specific biomarkers of epithelial functionality. These biomarkers included sulfomucin production and the gene expression of tight junction components and solute carriers. Notably, solute carrier 15 member 1 (SLC15A1), also referred to as oligopeptide (i.e., di-, tripeptides) transporter 1 (PEPT1) [44,45], is a low-affinity/high capacity transporter apically expressed on plasma membrane of mature enterocytes of all vertebrates, including pigs [46–49]. SLC15A1 serves as a marker of enterocyte maturation and is involved in peptide turnover and nutrient sensing [50,51]. Conversely, solute carrier 15 member 2 (SLC15A2, also known as oligopeptide transporter 2, PEPT2) is a high-affinity/low-capacity transporter of dietary di-/tripeptides, typically active in the distal intestine of vertebrates [47]. Additionally, the expression of tight junction protein 1 (*TJP1*, also known as zonulin 1 or *ZO-1*), a tight junction adaptor protein regulating adherens junctions, and occludin (OCLN), an integral membrane protein critical for cytokine-mediated modulation of tight junction paracellular permeability, serves as an indicator of gastrointestinal barrier integrity (see, e.g., [3–5]).

Beyond their effects on intestinal morphology and gene expression, dietary prebiotics and probiotics can influence the composition and diversity of the gut microbiota, which plays a critical role in immune development, nutrient metabolism, and epithelial barrier maintenance [52]. In particular, microbial communities in the colon are highly responsive to dietary interventions, and their modulation may contribute to host homeostasis through the production of short-chain fatty acids (SCFAs) and other bioactive compounds. Therefore, this study also aimed to assess microbiota profiles as an additional layer of intestinal functionality. The working hypothesis of this study posits that the combined administration of these pro- and prebiotic agents could enhance intestinal functionality and thereby promote piglet growth, supporting their potential as an effective synbiotic intervention.

## 2. Materials and methods

### 2.1 Ethical statement

The project has been approved by the Ministry of Health, Prot. n. 758/2020-PR, 27/07/2020.

### 2.2 Experimental design

#### 2.2.1 Sow management and feeding.

The study was carried out in a pig farm located in the Abruzzo Region (IT). Twenty crossbred pregnant sows (Large White × Landrace genetic lines) were involved in the study from day 107 of gestation until weaning piglets at 28 days age. All the animals received a basal diet formulated according to National Research Council (2012) [53] (NRC, United States); the ingredient composition and chemical analysis of the basal diet are given in Table 1. During the entire experimental trial, the pregnant sows were housed individually in farrowing pens (2.2 m × 2.4 m). The farrowing room was maintained at 20 °C throughout the experiment. The sows, homogeneous for parity, Body Condition Score (BCS) and farrowing date, were randomly assigned to one of the four diets (5 sow/group), as follows: CTR: basal diet without any supplementation; PRE: basal diet supplemented with 6 g/die of *Pichia guilliermondii* (PG) (CitriStim®, ADM Animal Nutrition, Quincy, IL); PRE/PRO: basal diet supplemented with 6 g/die of *Pichia guilliermondii* and 90 mg/die of *Bacillus velezensis* DSM 15544; PRO: basal diet supplemented with 90 mg/die of *Bacillus velezensis* DSM 15544 (BV) (Calsporin®, Miyarisan Pharmaceutical Co. Ltd). The experimental supplements were top-dressed on the gestation and lactation diet each morning to ensure consumption. Gestating sows were provided with a basal diet twice daily (at 08:30 and 14:30), totaling 2.5 kg per day. On the day of farrowing, sows were not fed and received 2 kg of feed the following day. Subsequently, feed intake was gradually increased by 1 kg per day until reaching free access to feed on the seventh day of lactation. During the experimental period, sows had *ad libitum* access to water. Pens were cleaned daily, regularly disinfected, and dewormed, while maintaining constant air circulation and stable temperature..

**Table 1:** Ingredient composition (%) of basal diet.

**Ingredients %**	
Yellow corn	28.5
Wheat bran	20.0
Wheat meal	18.5
Barley	10
Soybean seeds, 42.4% CP	6.5
Soybean meal, 53.5% CP	12
Dicalcium phosphate	1.2
Choline Chloride	0.18
Vitamin premix	0.2
Salt	0.3
Mineral premix	0.2
CaCO₃	1.7
Total	100
**Calculated composition (% as fed)**	
Dry matter	86.86
Crude protein	18.80
Crude fiber	3.80
Ether extract	4.10
Total Phosphorus	0.70
Total Lysine (g/kg as fed)	8.10
Total Methionine (g/kg as fed)	2.95
Total Cystine (g/kg as fed)	3.11
ME (Mcal/kg as fed)	3.33
DE (Mcal/kg as fed)	3.56

#### 2.2.2 Piglet management and feeding during lactation and post-weaning periods.

At birth, each piglet was individually weighed and tagged. Between 6 and 12 h after the birth of the last piglet, litter size was adjusted by cross fostering piglets within dietary supplementation groups to ensure that sows nursed a similar number of piglets (12 piglets/sow) throughout the entire lactation period. During this period, each piglet’s health status was monitored every day by specialized animal care personnel, while body weights (BW) were recorded at birth and 28 days after birth to calculate the average daily gain (ADG). No creep feed was offered to piglets throughout the lactation period, and they had no access to the sow’s feed. From farrowing until weaning (28 days after birth) all piglets orally received the same supplement as their dams using disposable pipettes following the four dietary supplementations as follows: CTR: water; PRE: 300 mg CitriStim®; PRE/PRO: 9 mg of Calsporin® + 300 mg of CitriStim®; PRO: 9 mg of Calsporin®.

At 28 days after birth, the piglets from each litter were grouped according to the feed supplementation and transferred into 4 weaning pens (60 animals per diet) with a space allowance of 0.70 m^2^/pig. Each pen had a temperature control system to achieve the desired thermoneutral zone for the animals and was equipped to give free access to feed and water.

From the day of weaning (28 days after birth) until 32 days post-weaning, all the animals received the same weaning starter feed each morning (09:00 h) with the addition, on the top of the feed, of the same supplementation received during the lactation period. The composition of weaning starter and the quantities of each supplement are shown in Table 2 (CTR: only starter feed; PRO: 30 mg/head/die of BV; PRE: 1 g/head/die of PG; PRE/PRO: 30 mg of BV + 1 g of PG/head/die). Piglets and feed offered were weighed on day 2 (T1), 17 (T2) and 32 (T3) post-weaning to determine the ADG and the average daily feed intake (ADFI). The intake was calculated on the total amount of feed distributed and the residue 24 hours later for each group.

**Table 2:** Formula and chemical composition of piglet weaned diet.

**Ingredients %**	
Yellow corn	20.88
Soybean meal, 53.5% CP	39.26
Whey powder	3.5
Choline Chloride	0.18
Lactose	9.8
Barley	24.11
Soy oil	0.43
Monocalcium phosphate	0.97
CaCO₃	0.98
DL-methionine	0.03
Vitamin premix	0.12
Mineral premix	0.12
Salt	0.2
Choline Chloride (25%)	0.12
ZnO	0.12
L-lysine HCl	0.33
TOTAL	100
**Calculated composition (% as fed)**	
Dry matter	89.45
Crude protein	22.80
Crude fiber	2.30
Ether extract	7.80
Total Phosphorus	0.85
Calcium	0.70
Sodium	0.33
Total Lysine (g/kg as fed)	1.72
Total Methionine (g/kg as fed)	0.62
ME (Mcal/kg)	3.58

### 2.3 Sample collection

At 2-, 17- and 32-days post-weaning, 6 piglets from each group were humanely euthanized inducing unconsciousness with azaperone (2 mg/kg i.m.) and ketamine (5 mg/kg i.m.) and immediately sacrificed with an overdose of sodium pentobarbital solution (60 mg/kg), followed by exsanguination [54].

Intestinal samples (approx. 15 cm of each tract) were dissected from the proximal duodenum, middle portion of jejunum, terminal ileum, cecum, and colon using sterilized instruments for each tract. Each segment was rinsed free of the intestinal content, and samples were collected for histology and gene expression analysis. Briefly, part of the tissues was immediately fixed in 10% neutral buffered formalin and stored at room temperature for 72 h for routine histology. For molecular analysis, tissues were collected in the manufacturing suggested amount of RNALater (ThermoFisher Scientific, Italy) and frozen at −80 °C. The remaining part of all tissues was also stored by freezing as a backup for possible further analysis. During sample collection, faecal score was evaluated in each piglet intestine following these criteria: 0 = normal feces, 1 = soft feces, 2 = mild diarrhea, 3 = severe diarrhea.

### 2.4 Examination of intestinal macro/micro morphology

#### 2.4.1 Gross pathology.

All the animals selected for the experimental protocol (72 selected piglets) were grossly examined for evidence of pathology. In particular, the presence of peritoneal exudate, lymph node enlargement, hemorrhages on the intestinal wall, splenomegaly and renal lesions were checked.

#### 2.4.2 Histopathology and villi morphometry.

Formalin-fixed intestinal segments were routinely processed for histology. Three cross-sections (4 µm) of each segment were obtained and stained with hematoxylin and eosin. The following variables were evaluated in 20 well-orientated villi per slide using a Leica DM3000 microscope and the imaging software LAS X version 02, LEICA (Leica Microsystems AG, Wetzlar, Germany): villus width (µm), villus height (µm), crypt height (µm), villus area I (µm²), villus area II (µm²), mucosal thickness (µm), villus perimeter (µm). Villus area I was evaluated with the aforementioned software, whereas villus area II was calculated with the formula 3.14*width*height according to [54,55]. As far as cecum and colon were concerned, 20 measurements of mucosal thickness were considered for each piglet.

#### 2.4.3 Sulfomucin-containing Goblet cell counting.

For quantification of sulfomucin-containing Goblet cells, sections were stained with Alcian Blue at pH 1.0 [56]. Sulfomucin positive cells were counted in 10 well-orientated villi and their corresponding crypts, which were randomly selected using the aforementioned microscopic system.

#### 2.4.4 Villi turnover analysis through cleaved-caspase-3 and Ki-67 immunohistochemistry (IHC).

Slides were subjected to IHC using a primary antibody directed against cleaved-caspase-3 (1:200 dilution, Abcam Ab4051) for the detection of apoptotic cells and Ki-67 (1:200 dilution, Dako, clone MIB1 code M7240) for proliferating cells. Briefly, sections were treated with citrate buffer solution 0.01 M pH 6.0 (cleaved-caspase-3) or EDTA buffer pH 9.0 (Ki-67) in a microwave oven for 15 min for antigen retrieval and with 5% (w/v) bovine serum albumin and 5% (v/v) normal goat serum for 15 min each to block unspecific binding sites. Labelling was subsequently detected using an ImmPRESS HRP Universal (horse anti-mouse/rabbit IgG) PLUS polymer kit (Vector Laboratories, CA, USA) with 0.1% (v/v) hydrogen peroxide in a 3,3’-diaminobenzidine solution (Millipore Sigma, St. Louis, MO, USA) as chromogen. Sections were finally counterstained with Mayer haematoxylin (Merck, Darmstadt, Germany).

Cleaved-caspase-3 and Ki-67 positive enterocytes were counted in 10 well orientated villi and corresponding crypts considering at least 1000 cells for each slide. Apoptotic and proliferation index were obtained and expressed through the percentage of positive cells on the total cell number for each villus.

### 2.5 mRNA expression analysis of epithelial functional marker genes

For mRNA expression analysis of genes coding for oligopeptide transporters (*SLC15A1* and *SLC15A2*) and junction proteins (*TJP1* and *OCLN*), total RNA was isolated using the AllPrep DNA/RNA/Protein Universal Kit (QIAGEN, Italy), according to manufacturer’s instructions. To obtain maximum quantity of RNA, < 50 mg of individual intestinal samples were cut, and preliminary steps of homogenization and lysis were performed. At the end of extraction, RNA aliquots were quantitatively and qualitatively checked using a Nanodrop ND-1000 spectrophotometer (Thermo Fisher Scientific, Italy). Electrophoresis of RNA samples was also performed in agarose gel (1% w/v) for checking genomic DNA contamination and/or RNA integrity. First-strand complementary DNA (cDNA) was synthesized from 500 ng of RNA using the iScript Select cDNA Synthesis kit (Bio-Rad, Italy) with random primers, according to manufacturer’s instructions. For each investigated gene, specific primers were designed spanning an exon-exon junction to avoid genomic amplicons using the Splign mRNA to genomic alignment tool (https://www.ncbi.nlm.nih.gov/sutils/splign/splign.cgi). The program AmplifiX (https://inp.univ-amu.fr/en/amplifx-manage-test-and-design-your-primers-for-pcr) was used to test PCR size, GC content, end stability and, self/cross-dimer formation of the selected oligonucleotides. All primer pairs were tested for primer efficiency. Sequences and detailed information of the used gene-specific primers are provided in Table 3. All qPCR reactions were performed using the iTAQ Universal SYBR Green Supermix kit (Bio-Rad, Italy) in a 20 µl final volume, with a CFX96 Touch Real-Time System (Bio-Rad) equipped with the CFX Manager Software (Bio-Rad). Melting curve analysis over a range of 65–95 °C (0.5 °C increment for 5 s) allowed for the detection of possible non-specific products and/or primer dimers. For relative quantitation, *hypoxanthine phosphoribosyltransferase 1* (*HPRT1*) gene was used as internal control (housekeeping). Relative gene expression was assessed by analyzing the output threshold values (Ct) according to the comparative Ct method [57]. qPCR data are shown as 2^-ΔCt^, which are taken as proportional to the amount of the detected target mRNA. ∆Ct values were obtained as target gene Ct – housekeeping gene Ct.

**Table 3:** Detailed information of primer sequences.

Gene	Accession Number	Primer sequences	Amplicon (bp)	T annealing (°C)
*HPRT1*	NM_001032376.2	Primer F: CTGAAGAGCTACTGTAATGACC Primer R: GTTGAAGATATAATTGACACTGGC	117	49
*SLC15A1*	NM_214347.1	Primer F: GGCTGTATCTCTGATTGTGTTT Primer R: AAGGAGAAATATGACGAGCGGCT	192	53
*SLC15A2*	NM_001097514.1	Primer F: TTGGATCAGCAGGGCTCAC Primer R: GACCAGATGCA**G**GTGTTAAACCCC	102	53
*TJP1*	XM_021098856.1	Primer F: AACAGAAATGTACAGCAAGGG Primer R: TATAGATCCAAAGAGGCCACG	142	53
*OCLN*	NM_001163647.2	Primer F: GCCTACTCGTCCAACGGGAA Primer R: TAATGAGGCTGCCTGAGGTCCT	134	59

### 2.6 Statistical analysis

Regarding micromorphology features, to evaluate the effects of the different dietary supplementations among groups (PRE, PRO, PRE/PRO) and relative to the control (CTR; non-supplemented), Kruskal-Wallis test was used for each post-weaning period after normality checking using Shapiro-Wilk test. “Time” was not considered as an independent variable, because the measured features were all physiologically time dependent. Using R software [58], a principal component analysis (PCA) was conducted to identify group distribution, and which morphometric variables or intestinal tracts were most representative of the experimental model. Additionally, multivariate linear regression was performed to determine the best predictors of piglet weight. The weights of the animals were analyzed using a mixed model (JMP Pro v18, SAS Institute), where diet and time were included as fixed effects. Individual piglets were considered the experimental units. When significant differences were detected (p < 0.05), means were separated using Tukey’s test. For gene expression and faecal score analyses, data (expressed as 2^-ΔCt^) were also tested for normality and equal variance using a Shapiro-Wilk test. Grubb’s outlier test was run prior to statistical evaluations. In order to assess possible significant effects, ordinary one-way ANOVA followed by a Dunnett’s multiple comparison test was used among the same intestinal segments of the different diets (* = *p* < 0.05; ** = *p* < 0.01; *** = *p* < 0.001; **** = *p* < 0.0001). All statistical analyses and graphs were produced using GraphPad Prism 8.2.1 (GraphPad Software, La Jolla, CA, United States).

### 2.7 Microbiota analysis

DNA amplicon library preparation was performed according to the Illumina 16S Metagenomic Library Prep Guide (15044223-b.pdf) targeting the V3-V4 hypervariable regions of 16S rRNA gene. First- and second- round PCRs were performed using SimpliAmp Thermal Cycler (Thermo Fisher Scientific, Waltham, MA), setting the thermal profiles according to the protocol. The first PCR amplified the target, while the second round PCR attached IDT UD indexes (Integrated DNA Technologies, Newark, NJ) to every single sample. Amplicon libraries were then pooled in a single tube and subjected to quality control using Qubit 2.0 fluorometer (Thermo Fiisher Scientific, Waltham, MA) and TapeStation 4200 (Agilent, Santa Clara, CA) to verify the concentration and size distribution, respectively. The pooled, indexed libraries were then denatured into single strands and diluted to 8 pM for sequencing with MiSeq v3 reagents (600 cycles) on an Illumina MiSeq instrument. PhiX Sequencing Control V3 was added at 20%. Raw sequencing data were provided in fastq format and analysed with the open-source bioinformatics pipeline QIIME 2, for quality control, taxonomic classification, and community composition assessment. Taxonomic identities of representative sequences were assigned by performing nucleotide similarity searches using BLAST against the NCBI database.

## 3. Results

### 3.1 The PRE/PRO group exhibits higher performance in terms of body weight

Table 4 shows the growth performance of piglets during the weaning period. At weaning (28 days after birth), the PRE group displayed a lower body weight (6536.15 g) in comparison to both PRO (7475.38 g) and PRE/PRO (8121.23 g) groups (*p* < 0.01), while it was similar to the weight of the CTR group (6796.35 g). The weight of the PRE/PRO group (8121.23 g) was also significantly higher (*p* < 0.01) on the weaning day when compared to the PRO group (7475.38 g). At 2, 17, and 32 days post-weaning (T1, T2 and T3, respectively), the PRE/PRO group consistently exhibited higher performance in terms of weight compared to the other three groups. In particular, at T1 the weight of the PRE/PRO group was higher compared to the PRO (*p* < 0.01), while CTR and PRE showed the lowest weight (6899.62 g and 6692.12 g, respectively). The ADG was significantly higher in the PRE/PRO group between T3 and T1 (*p* = 0.01).

**Table 4:** Growth performance of weaned piglets.

	CTR	PRE	PRO	PRE/PRO	SEM	P-value
**Weaned pig performance**						
Weaning weight (g)	6796.35 C	6536.15 C	7475.38 B	8121.23 A	280.4	0.01
Mortality at weaning	0.33	0.12	0.31	0.28	0.25	0.68
**Post Weaning weight (g)**						
T1	6899.62 C	6692.12 C	7613.46 B	8467.69 A	286.0	0.01
T2	9531.08 B	9595.35 B	9990.81 B	11197.25 A	494.37	0.05
T3	17935.71 B	17684.62 B	18335.71 B	19900.00 A	1143.29	0.05
**Average daily gain (g)**						
T2-T1	219.3	241.9	198.1	227.5	125.58	0.25
T3-T2	560.3	539.3	556.3	580.2	158.17	0.56
T3-T1	394.1 B	392.6 B	382.9 B	408.3 A	137.55	0.01

*Analysis of growth performance. The letters A, B, C represent differences among the groups. T1: weight of piglet 2 days post-weaning; T2: weight of piglet 17 days post-weaning; T3: weight of piglet 32 days post-weaning.

### 3.2 Evaluation of intestinal macro- and micromorphology

#### 3.2.1 Effects on gross pathology, histopathological features, and faecal score.

On macroscopic examination, no major pathological features were observed, particularly in the GI tract, except for a mild serofibrinous abdominal exudation detected in the following cases: nos. 8 and 11 (PRE-T1), nos. 15, 17, 18 and 19 (PRE/PRO-T1, which also showed focal bacterial colonies and neutrophilic aggregates within the lumen of cecal mucosal glands, on histologic examination), no. 28 (CTR-T2), nos. 32 and 34 (PRE-T2, in association with pale liver discoloration), no. 40 (in association with moderate mesenteric lymphadenomegaly), nos. 41 and 42 (PRE/PRO-T2). In addition, hyperemia and mesenteric lymphadenomegaly were observed in case no. 2 (CTR-T1), whereas a bilateral pale kidney discoloration was detected in case no. 61 (PRE/PRO-T3).

Similarly, no substantial histological variations were observed among cases in relation to the different dietary supplementations and times, except for changes in the villous morphology. At time T1, long and digitiform villi were detected in the duodenum and jejunum of control cases (CTR-T1), although they were shorter and squat in the ileum, similarly to prebiotic-supplemented (PRE-T1) cases. In prebiotic/probiotic-supplemeted (PRE/PRO-T1) cases, villi appeared to be broader at the apex, especially in the jejunum and ileum, with the latter tract showing villi with squat appearance and short/medium length, similarly to probiotic-supplemented (PRO-T1) cases. In addition, cellular desquamation and vacuolar changes were observed on the villus apex in the jejunum of control (CTR-T1) and probiotic-supplemented (PRO-T1) cases, in association with mild neutrophilic infiltration, although these findings were much less evident in the other intestinal tracts. Apical cell desquamation also appeared to be markedly reduced in all intestinal tracts of prebiotic/probiotic-treated (PRE/PRO-T1) cases, where it was only focally detectable. At T2, duodenal and jejunal villi of control (CTR-T2) cases resulted to be shorter and thicker than the corresponding tracts in prebiotic/probiotic-supplemented (PRE/PRO-T2) cases, also showing moderate apical desquamation in the jejunum, cecum and colon. Similarly, prebiotic-supplemented (PRE-T2) cases showed a better preservation of villous apex morphology when compared to controls (CTR-T2), except for cecum and colon, where moderate apical cell flaking was observed. In addition, mild villous apex edema and dilatation of chyliferous vessels were observed in the jejunum of control (CTR-T2) cases, whereas these findings were absent or only mildly detectable in all supplemented cases. At T3, control (CTR-T3) cases showed long villi, although with a flatter apex when compared to all supplemented cases, which exhibited a more pointed villous apex. In general, flaking was mild and reduced in comparison to T1, without differences between groups.

All samples showed capillary and venous congestion within villi, mild interstitial edema within the submucosa together with variable infiltration of mononuclear cells, especially lymphocytes, and mild to moderate numbers of eosinophilic granulocytes in the lamina propria and submucosa.

Small bacterial colonies, associated with mild neutrophilic exudation, were seen on the villous apex or within cecal crypts of CTR-T1 cases, in the PRE/PRO-T1 case no. 13, in PRO-T1 cases nos. 15 and 21, and in CTR-T2 case no. 26, although with no sign of bacterial invasion. As well, neutrophilic infiltration was generally scarce and only detectable in small aggregates within the crypts of large intestine in all cases. In addition, the presence of *Balantidium coli* was detected in the cecum of case no. 11 (PRE-T1), in association with edema, necrosis and superficial epithelial apoptosis. In case no. 48 (PRO-T3), the presence of several *Balantidium coli* was also observed in the lumen, within cells, as well as in the submucosa of the colon, in association with massive epithelial desquamation.

Regarding faecal score, only 2 episodes of severe diarrhea (faecal score = 3) and 3 mild diarrheas (faecal score = 2) were recorded at post-weaning, with no differences among groups. All the raw data related to faecal score are available in Supplementary Materials (see S1 Data).

#### 3.2.2 Prebiotic and probiotic supplements and their combination differently exert their effects on villi morphometric features and Goblet cell counting.

All the raw data related to morphometric analysis and Goblet cell counting are available in Supplementary Materials (see S1 Data). Villus dimensional features were measured in each intestinal tract, at each post-weaning period. The corresponding values (median ± interquartile range) are shown in S1, S2 and S3 Tables, at T1, T2 and T3, respectively. The post-*hoc* differences among groups are detailed in Table 5A–5D, where they have been grouped in the different diets.

**Table 5:** Villi morphometric features.

(a)									
Small intestine morphometric features	PREBIOTIC								
	Duodenum			Jejunum			Ileum		
	T1	T2	T3	T1	T2	T3	T1	T2	T3
Width (µm)		0.018							
Height (µm)				0.019		<0.001			
Crypt height (µm)						<0.001			
Area I (µm²)				0.005		0.012			
Area II (µm²)				0.016	0.024	0.003			
Mucosal thickness (µm)				0.028		<0.001			
Perimeter (µm)				0.006		<0.001			
Goblet cells		<0.001							0.003
**(b)**									
**Small intestine morphometric features**	**PREBIOTIC/PROBIOTIC**								
	Duodenum			Jejunum			Ileum		
	T1	T2	T3	T1	T2	T3	T1	T2	T3
Width (µm)				<0.001			0.044		
Height (µm)	0.004			0.034					
Crypt height (µm)	<0.001			0.024		0.041		0.003	
Area I (µm²)				<0.001	0.004		0.003		
Area II (µm²)				<0.001			0.013		
Mucosal thickness (µm)	<0.001							0.011	
Perimeter (µm)	0.025			<0.001	0.032		0.002		
Goblet cells	0.004	<0.001			<0.001				<0.001
**(c)**									
**Small intestine morphometric features**	**PROBIOTIC**								
	Duodenum			Jejunum			Ileum		
	T1	T2	T3	T1	T2	T3	T1	T2	T3
Width (µm)							0.036		
Height (µm)		<0.001		<0.001					
Crypt height (µm)		0.035				0.004			
Area I (µm²)		0.014		<0.001					
Area II (µm²)		<0.001		0.001			0.015		
Mucosal thickness (µm)		<0.001		0.004					
Perimeter (µm)		<0.001		<0.001					
Goblet cells		<0.001	0.034	0.023	0.013				0.001
**(d)**									
	**Large intestine morphometric features**			Cecum			Colon		
				T1	T2	T3	T1	T2	T3
**PREBIOTIC**	Mucosal thickness (µm)			0.048		0.006	0.009		<0.001
	Goblet cells				0.002				
**PRE/PRO**	Mucosal thickness (µm)					<0.001			<0.001
	Goblet cells				<0.001			<0.001	0.001
**PROBIOTIC**	Mucosal thickness (µm)			0.033		<0.001			<0.001
	Goblet cells				<0.001			<0.001	0.001

* Analysis of villi histology and mucin production. Significant differences in morphometric features determined by supplementations in each post-weaning period. p values were obtained with a non-parametric One-way ANOVA analysis of intestinal morphometric features of supplemented animals compared to the control at each time. Purple shading indicates an increase in morphometric feature values, with the gradation depending on p values; blue shading indicates a decrease in morphometric feature values.

As shown in Table 5, prebiotic (PRE; Table 5A) and probiotic (PRO; Table 5C) supplements, as well as their combination (PRE/PRO; Table 5B), exerted different effects on the various intestinal tracts and post-weaning periods. In particular, the PRE/PRO combination was associated with a significant increase in different villi morphometric features in the duodenum and the ileum at T1 compared to the control, including villus height (*p* = 0.004), crypt depth (*p* < 0.001), mucosal thickness (*p* < 0.001) and villus perimeter (*p* = 0.025) in the duodenum, and increasing villus width (*p* = 0.044), area I (*p* = 0.003), area II (*p* = 0.013) and perimeter (*p* = 0.002) in the ileum, suggesting possible beneficial effects for minimizing weaning stress on piglet intestine.

**Fig 1:**
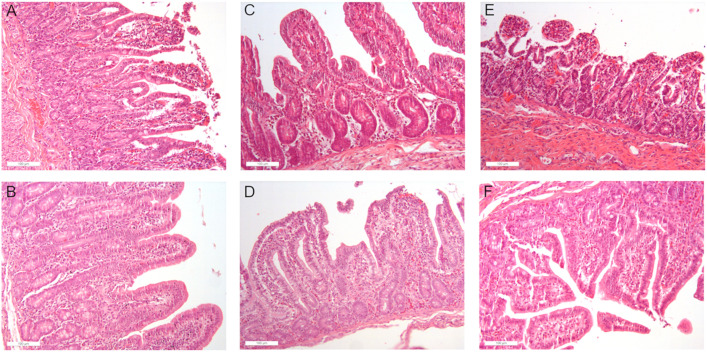
Morphology of the small intestine. Figures (A-F, 20x) show changes in intestinal morphology following PRE/PRO supplementation at T1. Specifically, in the duodenum [A (CTR) – B (PRE/PRO)] and in the ileum [E (CTR) – F (PRE/PRO)], a well-preserved villus structure and the absence of degenerative processes are evident in the intestine of the PRE/PRO supplemented group. In contrast, in the jejunum [C (CTR) – D (PRE/PRO)], villus fusion, reduced crypt depth, and apical degeneration are observed in the PRE/PRO supplemented group.

However, these positive effects at T1 were not evident in the jejunum, which exhibited a significant decrease in almost all villus morphometric features compared to the control (Table 5), suggesting a higher vulnerability of this intestinal tract to weaning stress, which could be difficult to overcome, even with dietary supplementation. On the other hand, the same tract at T3 revealed a positive sensitivity especially to the prebiotic supplementation. The beneficial effects of PRE/PRO supplementation in the duodenum and ileum at T1 and the lack of positive effects of the same supplemented diet in the jejunum are shown in Fig 1A–1F.

**Fig 2:**
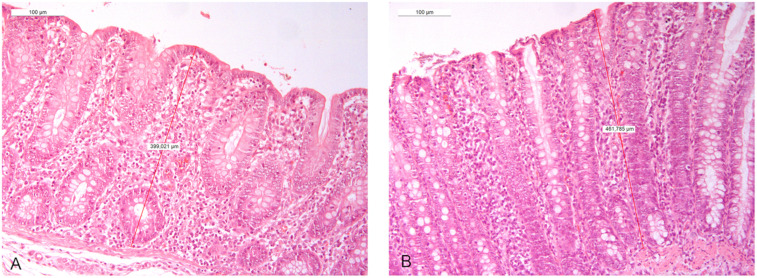
Colon mucosal thickness. Figures (A-B, 20x) show the increase in mucosal thickness following PRE/PRO supplementation at T3. Specifically, image A represents the CTR, image B the PRE/PRO supplemented group.

Regarding the large intestine, all dietary supplementations were associated with a significant increase in mucosal thickness, most notably at T3, both in the cecum and the colon (Table 5D). The increase in mucosal thickness associated with PRE/PRO supplementation is shown in Fig 2.

**Fig 3:**
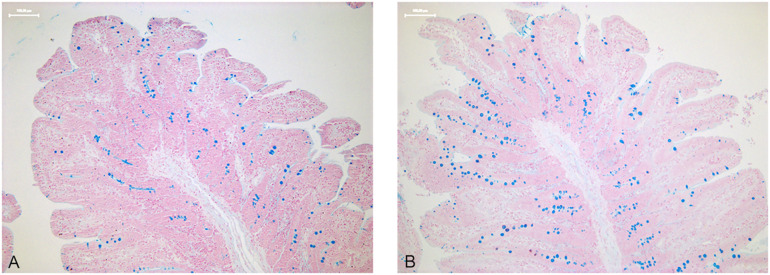
Goblet cell density in the jejunum. Figures (A-B, 10x) show the increase in Goblet cells density following PRE/PRO supplementation at T2 in the jejunum. Specifically, image A represents the CTR, image B the PRE/PRO supplemented group.

As reported in Table 5, the number of sulfomucin-containing Goblet cells in the duodenum at T1 did not change in the groups supplemented with the prebiotic or probiotic as single agents, whereas their combination was associated with a decrease in Goblet cell density. On the other hand, all supplementations, except for the prebiotic alone, resulted in a consistent increase in Goblet cells in the jejunum at T2, with the mixture showing the most pronounced effect. The increase in Goblet cells count in the jejunum associated with PRE/PRO supplementation is shown in Fig 3. At T3, a decrease in Goblet cell density was also observed across the small intestine, particularly in the ileum. In the large intestine, an increase in Goblet cells was noted at T2 in the cecum with all supplementations, as well as in the colon with both PRO and PRE/PRO supplementation. In contrast, a decrease in Goblet cell density was observed at T3 in association with PRO and PRE/PRO supplementation.

### 3.3 Villi morphometric features in the duodenum and the jejunum vary together in the experimental model

**Fig 4:**
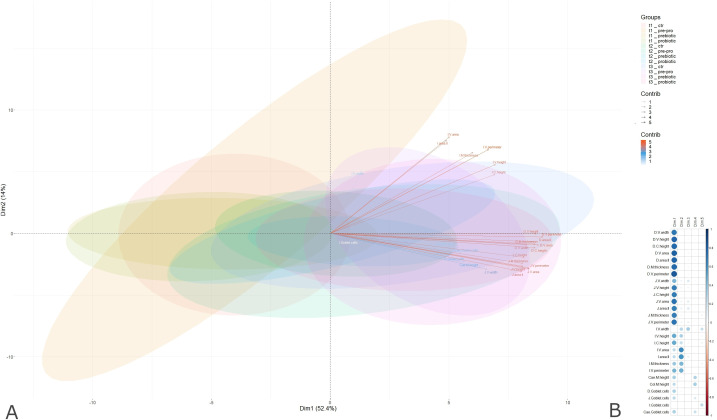
Correlation circles and correlation plot with groups and variables. (A) Correlation circles display how intestinal tracts represent the experimental model; arrow lengths and colors indicate the intensity of the contribution. (B) The correlation plot displays the quality of representation (cos^2^), where the color of the dots represents how much that variable contributes to one dimension, as shown in the legend. Intestinal tract names have been abbreviated as “D.V”: duodenal villi; “D.C”: duodenal crypts; “D.M”: duodenal mucosal; the same for the jejunum that is represented by the “J” and for the ileum that is the “I”. “Cae” indicates the cecum and “Col” the colon.

To understand which intestinal sites were most representative of the model while increasing the interpretability of results, we applied PCA. New uncorrelated variables were created, which maximize variance while minimizing potential information loss. The results showed that 52.4% of the variability was explained with two dimensions. Looking at the correlation circle (Fig 4A) and the correlation plot (Fig 4B), it is evident that some morphometric features vary together. The quality of representation of the variables can be expressed with the cos^2^. A high cos^2^ indicates a good representation of the variable on the principal component, whereas a low cos^2^ indicates that the variable is not perfectly represented by the principal components. It was found that villus height and width, crypt height, villus area and mucosal thickness of the duodenum and the jejunum are well represented by the first principal component, since the value of cos^2^ is close to 1. The same features related to other intestinal tracts, such as the ileum and the cecum, are represented by the first two dimensions, with minor contributions to the remaining dimensions (Fig 4A and 4B).

### 3.4 Improvements in duodenal, jejunal, and cecal morphology predict piglet weight

Linear regression analysis was performed to estimate the association between piglet weights and morphometric features, and to establish which of them predicted growth performance. The results indicated that the duodenum, jejunum, and cecum were the intestinal segments most strongly associated with increased piglet weight. In fact, a significant positive correlation with the weight was observed for duodenal villus width (*p* = 0.024) and jejununal villus height (*p* = 0.016). In the jejunum, villus perimeter was also positively correlated with piglet weight (*p* = 0.014). Finally, cecal mucosal thickness showed a positive correlation with piglet weights (*p* = 0.018). Correlation coefficients and *p* values are shown in S4 Table.

### 3.5 Dietary supplementation decreases apoptosis in the duodenum and the jejunum

**Fig 5:**
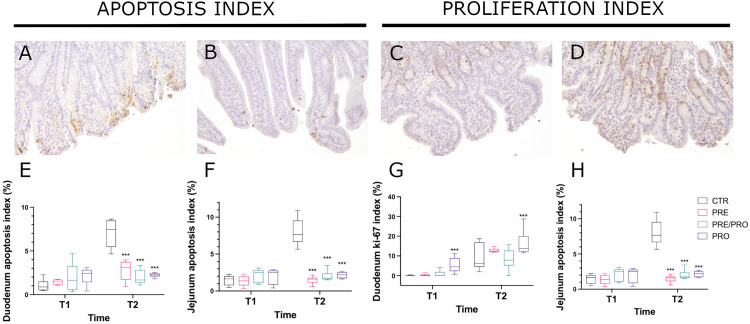
Mucosal turnover in the duodenum and the jejunum following different diets at T1 and T2 post-weaning periods. IHC images (top row) showing a significant decrease in the number of cleaved-caspase-3 positive cells observed at T2 in the jejunum following PRE/PRO supplemented diet (B_20x) and an increase in Ki-67 positive cells following PRO supplementation (D_20x), compared to CTR (A and C, respectively_20x). Box-and-whiskers plots reveal the median with the interquartile range of apoptosis index in the duodenum (E) and in the jejunum (F) and the proliferation index in the duodenum (G) and in the jejunum (H) for each group. (* = p < 0.05; *** = p < 0.001).

All the raw data related to mucosal turnover are available in Supplementary Materials. Based on the aforementioned PCA results, cleaved-caspase-3 and Ki-67 immuneexpression were evaluated in the most representative tracts of this model (duodenum and jejunum) to understand if the differently supplemented diets may influence mucosal turnover. The corresponding values (median ± interquartile range) are shown in S1 and S2 Tables, at T1 and T2, respectively. The post-*hoc* differences among groups are reported in detail in Fig 5, where they have been grouped in the different diets. The most evident results were represented by a decrease in the percentage of cleaved-caspase-3 positive cells at T2 (*p* < 0.001 for all the supplemented diets) both in the duodenum and the jejunum, as well as a significant increase in Ki-67 positive cells after PRO supplementation at T1 and T2 (*p* < 0.001) (Fig 5).

### 3.6 mRNA-expression analysis of epithelial functional marker genes

#### 3.6.1 PRE/PRO combination increases *TJP1* gene expression in the jejunum at 17 days post-weaning.

**Fig 6:**
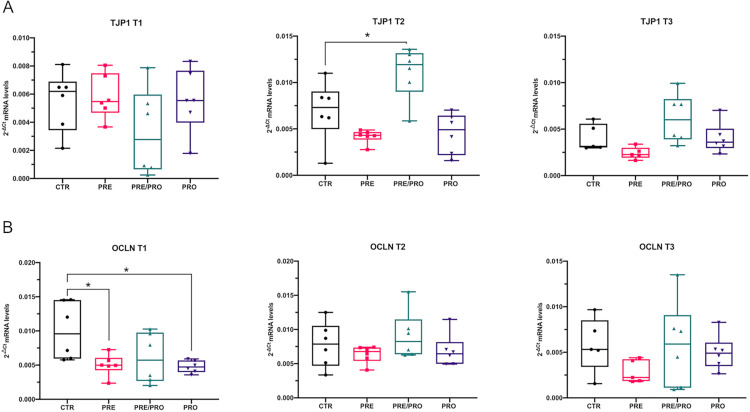
mRNA-expression levels of tight junction proteins in jejunal mucosa of weaned piglets fed with 4 different diets (CTR, PRE, PRE/PRO and PRO) at 3 different time points (T1, T2, T3). Values are represented as box plots (n = 6) of 2^-ΔCt^ normalized to *HPRT1* housekeeping gene. Statistical differences were assessed by an ordinary one-way ANOVA followed by post-hoc Dunnett’s test for multiple comparisons. Significance levels are denoted as: * = p < 0.05. *TJP1*: Tight Junction Protein 1, *OCLN*: occludin.

The results of the intestinal morphometry indicated that villus morphometric features (in terms of width and height) in the jejunum were predictive of piglet weight. Consequently, further investigations concerning possible molecular changes resulting from diet supplementations were focused on this intestinal tract. In particular, the mRNA expression of two genes involved in the formation of tight junctions (i.e., *TJP1* and *OCLN*) was analyzed. Our results showed that only PRE/PRO supplementation significantly induced upregulation of *TJP1* mRNA at T2 (*p* < 0.05) (Fig 6A). Conversely, a decreased expression of *OCLN* mRNA was observed with PRE and PRO supplementation at T1 (*p* < 0.05) (Fig 6B). All the raw data related to gene expression are available in Supplementary Materials (see S1 Data).

#### 3.6.2 Prebiotic and probiotic supplements and their combination differently exert their effects on *SLC15A1* and *SLC15A2* mRNA expression along the piglet intestine.

**Fig 7:**
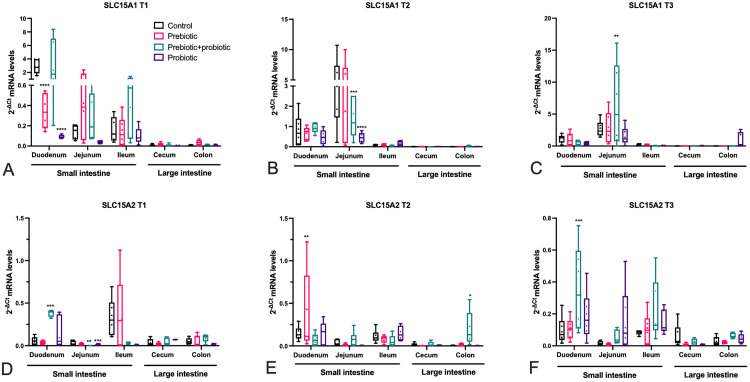
(A-F) Analysis of mRNA expression levels of *SLC15A1* (A-C) and *SLC15A2* (D-F) along all the intestinal segments of piglets fed with the four different diets at three different time points. Values are represented as box plots (n = 6) of 2^-∆Ct^ (normalized to *HPRT1* as the housekeeping gene). Statistical differences compared to the control group were assessed by an ordinary one-way ANOVA followed by a Dunnett’s multiple comparison test (* = p < 0.05; ** = p < 0.01; *** = p < 0.001; ****p < 0.0001).

Gene-expression data of the oligopeptide transporters are shown in Fig 7. Comparing the two rostro-caudal expression pattern along the various intestinal segments (duodenum, jejunum, ileum, cecum and colon) of piglets, it was noticed that *SLC15A1* showed higher mRNA expression levels in the anterior part of the intestine (duodenum, jejunum and ileum) followed by a remarkable decrease in the distal regions (cecum and colon) (Fig 7A–7C). On the other hand, *SLC15A2* mRNA expression did not show a specific trend, with low mRNA expression levels variably distributed along the intestinal segments (Fig 7D–7F). In general, *SLC15A1* mRNA levels were found to be higher than *SLC15A2* (Fig 7A–7F). For both genes, these general trends are in line with what is known in the literature about them being considered rostro-caudal physiological markers along the GI tract of all vertebrates [49]. More in detail, at T1, PRE and PRO diets used alone were associated with a significant (p < 0.0001) decrease in *SLC15A1* expression levels in the duodenum compared to CTR, whilst no significant changes were detected with the PRE/PRO combination (Fig 7A). At subsequent time-points (T2 and T3), significant changes were found in the jejunum; while the PRE diet was found ineffective on *SLC15A1* mRNA expression, the PRE/PRO diet was associated with significant changes of *SLC15A1* expression compared to CTR at T2 (down-regulation; p < 0.001) as well as at T3 (up-regulation; p < 0.01). Also, the PRO diet was associated with a down-regulated expression compared to CTR (p < 0.0001) at T2 (Fig 7B and 7C). Overall, *SLC15A1* mRNA expression demonstrated a specific sensitivity to PRE/PRO treatment in the jejunum across the time points, while duodenal responsiveness was found only at the early T1 time point.

Supplemented diets mostly affected *SLC15A2* mRNA expression in the duodenum and jejunum. In particular, PRE/PRO diet at T1 was associated with a significant up-regulation (p < 0.001) and a significant down-regulation (p < 0.01) compared to CTR in the duodenum and the jejunum, respectively, whereas PRO diet was related to a significant down-regulation (p < 0.001) compared to CTR only in the jejunum (Fig 7D). At T2, PRE diet was associated with a significant up-regulation (p < 0.01) compared to CTR only in the duodenum (Fig 7E), while PRE/PRO diet was associated with a significant upregulation (p < 0.05) of *SLC15A2* within the colon, but not in the small intestine (Fig 7E). Finally, at T3, the duodenum was the only intestinal tract showing significant changes in the mRNA levels with the PRE/PRO diet (upregulation, p < 0.001) compared to CTR (Fig 7F). Overall, the analysis of *SLC15A2* mRNA expression indicates that increasing levels mainly occur in the duodenum, with the PRE/PRO diet (at T1 and T3) or with the PRE diet (at T2). All the raw data related to gene expression are available in Supplementary Materials (see S1 Data).

### 3.7 Microbial composition

#### 3.7.1 α- and β-diversity.

**Fig 8:**
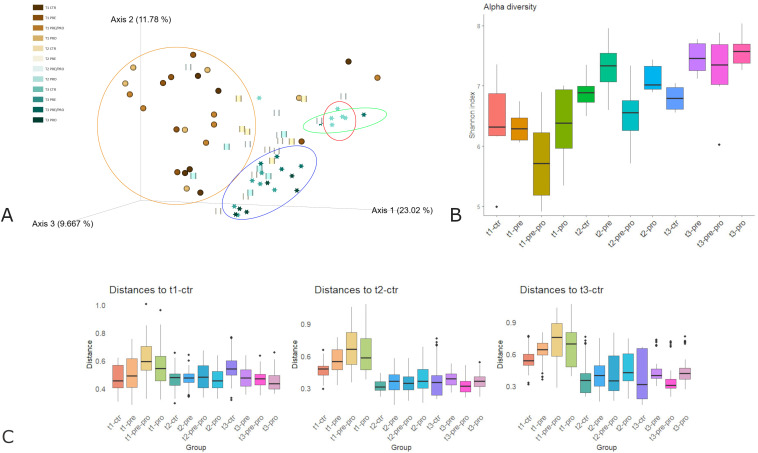
(A-C) α-diversity and β-diversity. Principal Coordinates Analysis (PCoA) plot based on Weighted UniFrac distances showing clustering of microbial communities (A). α-diversity (Shannon Index) across experimental groups, indicating differences in microbial richness and evenness (B). PERMANOVA results confirming statistically significant differences in community composition (β-diversity) between groups (C).

To account for both richness and evenness, α-diversity was assessed using the Shannon index. A trend of increasing diversity from T1 to T3 was observed across all groups, reflecting microbiota maturation. Statistical comparison revealed significant differences in α-diversity between supplemented groups and the control group at T3 (CTR *vs* PRE: *p value* = 0.016; CTR *vs* PRE/PRO: *p value* = 0.181; CTR *vs* PRO: *p value* = 0.016) (Fig 8A and 8B). Regarding β-diversity, Principal Coordinate Analysis (PCoA) based on the Weighted UniFrac distance revealed a clear clustering pattern especially at T3 between the CTR and the supplemented groups (PRE and PRO), while the PRE/PRO group showed increased dispersion, with a subset of samples clustering closer to the CTR group. PERMANOVA results confirmed that time explained the largest portion of the variance observed (Fig 8C).

#### 3.7.2 Differential abundance analysis (ANCOM).

**Fig 9:**
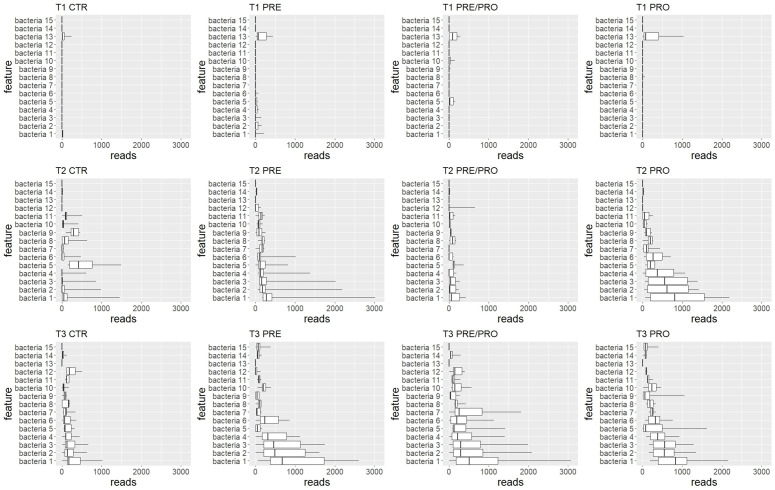
Differential taxonomic composition of the gut microbiota among experimental groups identified by ANCOM. Bacterial taxa showing significant differences are listed on the y-axis, with higher relative abundance observed in the supplemented groups.

ANCOM was used to identify differentially abundant taxa across groups and time points. Box plots were used to visualize the distribution of read counts per taxon across all supplemented groups and time points; only a few significant features were found at T1, while more differences emerged from T2 onward, mostly driven by temporal changes. (Fig 9). Bacteria belonging to the family *Lachnospiraceae* showed low abundance at T1 across all groups. A clear increase was observed at T2 in the PRE and PRE/PRO groups, with further expansion at T3. In contrast, T3 controls remained similar to T2, confirming that the variation was diet-driven (Fig 9 and Table 6). *Phocaeicola vulgatus*, which had the highest W score, exhibited a different pattern (Fig 9 and Table 6): it was abundant at T1, but decreased consistently in supplemented groups over time.

**Table 6:** Differentially abundant taxa of gut microbiota.

	W*	Species
Bacteria 1	3802	*Lachnospiraceae* sp.
Bacteria 2	3624	*Lachnospiraceae* sp.
Bacteria 3	3682	*Lachnospiraceae* sp.
Bacteria 4	3515	*Lachnospiraceae* sp.
Bacteria 5	3563	*Alloprevotella* sp.
Bacteria 6	3597	*Lachnospiraceae* sp.
Bacteria 7	3729	*Agathobacter rectalis*
Bacteria 8	3464	*Faecalibacterium prausnitzii*
Bacteria 9	3606	*Alloprevotella* sp.
Bacteria 10	3434	NA (uncultered bacterium)
Bacteria 11	3653	*Faecalibacterium prausnitzii*
Bacteria 12	3630	*Oscillospiraceae* sp.
Bacteria 13	3819	*Phocaeicola vulgatus*
Bacteria 14	3447	*Prevotella hominis*
Bacteria 15	3671	NA uncultured bacterium

*W indicates the number of pairwise log-ratio comparisons in which a given taxon was found to be significantly different.

## 4. Discussion

In intensive farming systems, piglets are typically weaned early, at 3–4 weeks of age, compared to the natural weaning age of approximately 10–17 weeks [2]. Early weaning aims to improve sow productivity but exposes piglets to stress that negatively affects their growth, health, and intestinal function [59]. In the last decades, various scoring systems have also been employed to assess intestinal histological parameters, with or without gut inflammation, in experimental settings involving dietary supplementation [31,60,61]. For this reason, growth performance, as well as intestinal morphology and functionality, were selected as valuable endpoints to investigate the effects of diets supplemented with specific pre- and/or probiotic (alone or in combination) on post-weaning piglets. The growth performance during the post-weaning period, from the beginning (28 days post-farrowing) until 32 days post-weaning showed clearly a better response with the combined experimental diet (PRE/PRO) compared to the three other groups.

In addition, the analysis focused on the impact of the supplemented diets on intestinal morphology (in terms of gross pathology and morpho-histological features, principal component analysis and linear regression) and functionality (i.e., mucosal turnover and transcriptional expression of markers of peptide transmembrane absorption/transport and paracellular permeability), by evaluating their differential effects on both the small (duodenum, jejunum and ileum) and large intestine (cecum and colon).

### 4.1 Evidence from small intestine in piglets receiving different dietary supplements

To better interpret the effects of different dietary supplements, the control group trends during the post-weaning period were first examined. Villus size consistentlyincreased over time, particularly at T2 and T3 compared to T1. This improvement, along with the absence of typical post-weaning clinical signs, suggests that good farm management effectively reduced piglet stress in this study. In fact, during our study, only 2 episodes of severe diarrhea (faecal score = 3) and 3 mild cases of diarrhea (faecal score = 2) were recorded at post-weaning, with no differences among groups, an aspect probably related to the optimal rearing and experimental conditions ensuring animal health.

Notwithstanding this, as far as the effects of the differently supplemented diets were concerned, an earlier (i.e., at T1 = 2 days post-weaning) improvement of villi dimensional features associated with the PRE/PRO supplementation was observed in both duodenum and ileum, although it was not similarly evident in the jejunum, which showed substantial alterations of villus morphology regardless of supplementation, suggesting a higher vulnerability of this tract to the post-weaning stress. In particular, at the jejunal tract, the PRE/PRO supplementation was associated with a restored morpho-functional state only at T2 (17 days post-weaning), whereas the PRE-supplementation alone only at T3 (32 days post-weaning).

The greater jejunal sensitivity to early post-weaning stress (T1), despite supplementations with all the experimental diets, was possibly due to the high mucosal turnover usually observable in this tract, even in normal conditions. In fact, differently from duodenum and ileum, active extrusion zones can already be found in the jejunum of piglets at one week of age [62,63]. The jejunal stress revealed by villus morphological alterations was also confirmed at the molecular level with a significant decrease of *OCLN* mRNA expression at T1 in this tract, since occludin downregulation may promote epithelial survival during stressful events [64]. Likewise, the upregulation of *TJP1* mRNA levels is in line with a possible restoration of villus functionality induced by PRE/PRO supplementation at T2, manifested by increased villus area and perimeter [65].

A higher jejunal vulnerability to the initial phase of the post-weaning period is also in agreement with the results obtained in the study of Michiels et al. [41] using *Bacillus velezensis* DSM 15544 on sows and sucklings. Pre-weaning supplemented piglets had lower villus height at day 0 but showed similar values to other groups after weaning, suggesting they experienced less villus atrophy and adapted better to weaning. A gradual villus height decrease during suckling may indicate enhanced gut maturation in this group. Similarly, van der Peet-Schwering et al. [66] did not find any effects of yeast cell walls on jejunal morphology in piglets.

Most of the digestion and nutrient absorption typically occurs in the small intestine: while the duodenum is predominantly the site where food and pancreatic secretions are mixed, the jejunum acts as the major site of absorption [60]. These aspects are in line with our results, in which increased villus dimensions in duodenum and jejunum were correlated with piglet weights, as shown in the linear regression. In particular, the greatest increase in piglet weights was observed at T1 with PRE/PRO and PRO supplementations, and partially at T2 with only PRE/PRO supplementation. As well, at two days post-weaning (T1), the combined supplementation (PRE/PRO) led to a significant improvement in villus morphometry in both duodenum and ileum, which could compensate jejunal stress in term of absorption, since a positive synbiotic effect in the jejunal tract was only observed at T2. Recently, Sandrini et al. [43] observed an increase in villus height, villus width, and crypt depth in the ileum of piglets supplemented with a mixture containing *Pichia guilliermondii* 28 days after weaning. Interestingly, we observed similar changes in the jejunum, using the prebiotic alone at the same post-weaning period, whereas in the ileum, an improvement of villus dimensional features was already detectable 2 days after weaning using our combined supplementation. The supplementation of both pre-weaning piglets and sows during the lactation period should be considered as a contributing factor to these results in our experimental model.

Post-weaning changes in intestinal tissues, including alterations in villus, crypt architecture and destruction of tight junctions have been well documented [67]. Villus atrophy following weaning is caused by an increased rate of cell loss (apoptosis), as well as a reduced rate of cell renewal (mitosis), which is highly pronounced in the duodenum and ileum. At the same time, it has been previously observed that tissue recovery is physiologically more pronounced in the duodenum starting from 3 to 14 days post-weaning, thus suggesting a higher cellular turnover in the proximal small intestine in that period [30,39,68]. This evidence aligns with our results, explaining the fast recovery of duodenum and ileum after post-weaning stress at T1 in association with PRE/PRO supplementation, together with a consistent post-weaning decrease of apoptotic cells (cleaved-caspase-3 positive cells) at T2 (17 days post-weaning) in the duodenum of animals fed with supplemented diets, compared to the control group.

Moreover, in the present study, sulfomucin-containing Goblet cells in the duodenum at T1 were not affected by the prebiotic or probiotic used as single agents, whereas their combination led to a decrease in Goblet cell density. On the other hand, at T2, all supplementations, except for the prebiotic alone, induced a consistent increase of Goblet cells in the jejunum, with the mixture having the most notable effect. Then, at T3, a decrease in Goblet cell density was observed across the small intestine, particularly in the ileum. The concentration of sulfomucins increases together with the maturation of the functional barrier of newborn piglets and rat intestine [67,69,70]. During early development, innate immunity is crucial due to the immaturity of the acquired immune system. [71]. In this study, no signs of intestinal infection were found. At T3, supplemented groups showed reduced Goblet cell density, possibly reflecting a more mature epithelium requiring less mucus production. Control piglets had higher sulfomucin-producing Goblet cells in the ileum, although sulfomucin levels were not elevated at T3.

PCA analysis revealed that most diet-related structural changes were concentrated in the duodenum and jejunum. Traits like villus height, width, crypt depth, mucosal thickness, and area I and II were strongly associated with the first principal component and body weight. In particular, villus height and perimeter showed significant correlations with growth, suggesting that these intestinal adaptations support improved nutrient processing during the weaning transition [72]. Linear regression further confirmed that structural changes in these intestinal segments were significantly linked to increased body weight. Accordingly, gene expression data of the *SLC15A1* peptide transporter were also indicative of diet-specific effects in the duodenum and jejunum. Moreover, these effects appeared to be dependent on the T1-to-T3 progression. It is worth noting that the PRE/PRO supplemented diet significantly increased *SLC15A1* expression in the jejunum when compared to control diet at T3, a finding suggesting that the absorption of peptide sources (i.e., deriving from digestion and/or protein turnover) may find its physiological “steady-state” after long-term administration. Overall, since *SLC15A1* is a marker of functional maturation of the absorptive epithelium [73] it must be considered that its expression fluctuations based on the type of diet, as well as on the sampling time, may depend on multifactorial conditions including *i*) an accelerated turnover and/or maturation of enterocytes, *ii*) the accelerated or not accelerated demolition of mucins, *iii*) the remodeling of villi and microvilli, and/or several other factors [74,75]. Similar to *SLC15A1*, the differently supplemented diets exerted some effects on *SLC15A2* mRNA expression. It is interesting to note that, in the overall picture of time-dependent or diet-dependent trends, *SLC15A2* usually results to be regulated in a compensatory manner compared to *SLC15A1* [76]. Considering the synergistic role of these two transporters on the intestinal epithelium, these complementary trends may indicate the occurrence of an epithelial physiological response in all conditions involving either a massive presence or a lower availability of oligopeptides. The hypothesis of a multifactorial effect on *SLC15A1*/*SLC15A2* modulation is supported by linear regression analysis since no significant correlations were found to support the predictive role of these two gene expressions.

### 4.2 Evidence from large intestine in piglets receiving dietary supplements

In the large intestine (cecum and colon), only mucosal thickness was evaluated due to the lack of villi. A general increase in mucosal thickness was observed at T3 across all supplemented diets, suggesting a positive impact. Since the large intestine is key for water and mineral absorption and immune regulation, this thickening may benefit piglet health [77]. Additionally, a reduction in sulfomucin-producing Goblet cells at T3 might indicate improved development of endocrine and immune functions. [78]. As well, the absence of clinicopathological signs (e.g., diarrhea, catarrhal enteritis) and altered expressions of *SLC15A1* and *SLC15A2* also appears to be in line with this hypothesis. Regarding the PCA analysis, the cecum tract contributed less strongly to the experimental model, but linear regression revealed a significant positive correlation between cecal mucosal thickness and piglet weight. This suggests that the cecum may play a complementary role in growth, potentially through improved barrier function or enhanced microbial fermentation [79].

Finally, the present study detected the presence of *Balantidium coli* within the cecum of two piglets (no. 11 and 44). This parasite inhabits the large intestine of several animals other than pig, which can trigger various GI manifestations in the infected hosts, such as diarrhea with mucus and blood [80,81]. Although the histological analysis revealed various pathological effects associated with the presence of this protozoon (i.e., invasion of the apex of the villi, apical cell necrosis and mild inflammatory infiltration), they did not have a clinical impact, being the animals only focally and mildly parasitized.

### 4.3 Gut microbiota and synergistic mechanisms of *Bacillus velezensis* and *Pichia guilliermondii*

Gut microbiota profiling revealed a progressive increase in α-diversity across all groups, consistent with post-weaning maturation [79]. Animals fed with all the different diets showed enhanced microbial richness and distinct β-diversity patterns at later time points [82]. Notably, members of the Lachnospiraceae family, known for producing SCFAs such as butyrate, were enriched in supplemented groups. These microbial shifts correlate with the histological and molecular findings in the colon, where dietary supplement enhanced mucosal integrity. The presence of SCFA-producing bacteria may contribute to tight junction maintenance and reduced inflammation [83]. These findings underscore the interplay between microbial composition and host response, highlighting the potential of microbiota-directed strategies to reinforce colonic health in weaning piglets.

The synergistic mechanisms of *Bacillus velezensis* and *Pichia guilliermondii* supplementation likely rely on a combination of direct microbial actions and modulation of host responses. B. velezensis produces a wide array of antimicrobial lipopeptides, which inhibit pathogenic bacteria and favor the establishment of beneficial commensals [84]. Moreover, its ability to produce enzymes (e.g., proteases, amylases) supports nutrient digestion and absorption [85]. In parallel, the yeast-derived cell wall components of *P. guilliermondii*, particularly β-glucans and mannanoligosaccharides (MOS), exert immunomodulatory effects by binding pattern recognition receptors on mucosal immune cells, enhancing innate immune readiness [86]. This combination of microbial metabolite production, immune stimulation, and microbiota modulation likely underlies the observed improvements in gut morphology and function in supplemented piglets.

### 4.4 Limits of the study

This study offers important insights into how *Pichia guilliermondii* and *Bacillus velezensis* supplementation supports intestinal health and growth in post-weaning piglets. However, its limitations include being conducted on a single pig breed under controlled conditions, which may limit generalizability to broader commercial settings. Nonetheless, the study was performed in a commercial farm context reflecting regional industry standards, and the controlled design helped improve animal health and welfare. By combining zootechnical, pathological, and biomolecular evaluations, the study gives a more complete picture of how such supplements influence piglet physiology. Based on the findings, the use of functional nutritional strategies like prebiotics and probiotics is recommended to reduce antibiotic use and boost immunity during weaning. Furthermore, the adoption of standardized welfare monitoring protocols, including behavioral, clinical, and molecular indicators, is encouraged to improve welfare outcomes and align with current regulations. Finally, while key molecular markers were analyzed, further investigation into additional immune-related factors is ongoing.

## 5. Conclusions

Taken together, these findings provide a coherent picture of how dietary supplementation in early life can modulate gut structure and function in a region-specific manner. The duodenum and jejunum clearly emerge as the primary sites of adaptation, showing both structural changes and functional shifts in cell turnover. The cecum, although secondary, appears to offer additional support, possibly by improving fermentation efficiency or mucosal resilience. The combined use of PCA and regression modeling allowed us to pinpoint which features were both statistically significant and biologically meaningful. In conclusion, pre- and probiotic (PRE/PRO) supplementation appears to promote intestinal health by enhancing villus structure, reducing epithelial loss, and stimulating proliferation, particularly in the proximal small intestine. These changes are not merely morphological but likely reflect deeper functional adaptations that support nutrient uptake and overall piglet performance during the critical post-weaning window.

## Supporting information

S1 Table Median ± interquartile range of villi dimension features at t1. (DOCX)

S2 Table Median ± interquartile range of villi dimension features at t2. (DOCX)

S3 Table Median ± interquartile range of villi dimension features at t3. (DOCX)

S4 Table Linear regression analysis. (DOCX)

S1 Data Raw data. (XLSX)
