# Supplementary material for: Dietary supplementation with Bacillus velezensis and Pichia guilliermondii improves growth performance through intestinal morphology and functionality enhancement in weaning piglets
Source: PLoS One. 2025 Dec 4;20(12):e0332920. doi: 10.1371/journal.pone.0332920 (PMC12677519; doi:10.1371/journal.pone.0332920)
Supplement: S1 Table — (DOCX) [file pone.0332920.s001.docx]

**Supplementary Table S1: Median ± interquartile range of villi dimension features at t1**

|  |  | **t1** | | | |
| --- | --- | --- | --- | --- | --- |
|  |  | **ctr** | **pre** | **pre/pro** | **pro** |
| **duodenum** | **villus width (µm)** | 152.95[119.73,175.63] | 150.18[106.74,195.65] | 146.01[116.15,187.62] | 147.94[116.75,174.11] |
|  | **villus height (µm)** | 509.12[441.14,594.59] | 585.48[495.09,661.09] | 539.7[451.42,605.71] | 487.64[377.02,577.7] |
|  | **crypt height (µm)** | 264.56[238.3,296.81] | 292.34[253.05,345.31] | 279.21[224.08,340.25] | pro+G2 |
|  | **villus area (µm2)** | 67731.64[51227.01,82116.27] | 74591.81[53359.99,101201.72] | 65410.78[50219.87,88355.21] | 61438.18[41699.84,81919.9] |
|  | **area II (µm2)** | 246313.33[192268.34,310304.45] | 272261.38[184514.72,377022.7] | 236837.87[163149.25,333952.67] | 224470.7[147739.63,293695.1] |
|  | **mucosal thickness** | 137112.43[106000.78,173780.91] | 171914.99[131253.18,221764.45] | 153071.68[100378.69,208997.82] | 132255.68[84473.56,170724.82] |
|  | **villus perimeter (µm)** | 1264.76[1118.27,1463.28] | 1429.54[1193.68,1595.05] | 1305.3[1134.21,1486.26] | 1209.41[1000.4,1426.27] |
|  | **Goblet cells** | 22[13,33.25] | 15[10,21] | 21[14,27] | 24[13,35.25] |
|  | **Cleaved-caspase-3 positive cells %** | 0.86[0,1.8] | 1[0.75,1.92] | 1.1[0,3.06] | 1.77[0.8,3.32] |
|  | **Ki-67 positive cells %** | 0[0,0] | 0[0,0] | 0[0,1.44] | 4.16[2.5,6.38] |
| **jejunum** | **villus width (µm)** | 128.55[109.36,164.58] | 115.12[92.87,137.18] | 129.53[103.41,150.98] | 127.11[103.01,158.15] |
|  | **villus height (µm)** | 426.31[363.17,496.21] | 393.95[349.56,451.13] | 392.26[321.19,452.99] | 340.47[296.41,426.39] |
|  | **crypt height (µm)** | 193.52[155.47,242.6] | 222.46[189.97,250.9] | 181.71[152.91,219.43] | 190.27[164.93,215.49] |
|  | **villus area (µm2)** | 49978.53[38434.39,70724.37] | 39582.66[32548.08,49860.31] | 42697.79[31358.19,55545.15] | 39490.85[27847.33,53417.23] |
|  | **area II (µm2)** | 183848.71[133794.3,255803.72] | 140561.05[106096.03,174465.33] | 151939.5[112049.65,201185.87] | 141708.62[100411.04,207883.82] |
|  | **mucosal thickness** | 83594.99[59892.14,118710.49] | 86527.44[68981.28,106256.12] | 66242.94[49809.74,101351.87] | 64398.89[52674.73,88619.16] |
|  | **villus perimeter (µm)** | 1102.06[950.88,1220.07] | 970.42[871.7,1092.25] | 977.4[852.71,1152.43] | 907.25[756.51,1081.83] |
|  | **Goblet cells** | 23[16,30.75] | 21[17,28.25] | 22[15,31] | 18.5[13,24.25] |
|  | **Cleaved-caspase-3 positive cells %** | 1.09[0.55,2.26] | 1[0.6,1.96] | 1.91[0.96,3.14] | 1.9[0,3.04] |
|  | **Ki-67 positive cells %** | 0[0,0] | 0[0,0] | 0[0,0] | 4.87[3.31,7.94] |
| **ileum** | **villus width (µm)** | 183.33[118.56,255.44] | 223.15[139.97,305.59] | 159.63[119.59,226.91] | 148.31[112.52,212.02] |
|  | **villus height (µm)** | 359.5[310.2,456.9] | 407.36[327.26,500.42] | 384.59[318.86,486.57] | 349.27[305.7,411.42] |
|  | **crypt height (µm)** | 173.98[139.73,215.85] | 184.36[143.6,274.49] | 189.37[148.22,228.47] | 193.92[161.21,222.63] |
|  | **villus area (µm2)** | 56913.28[35625.18,84163.7] | 71853.88[48174.33,103414.18] | 51645.63[36994.73,72934.77] | 48689.35[31939.59,63029.84] |
|  | **area II (µm2)** | 212365.28[127734.46,323484.74] | 265298.09[178176.15,400538.6] | 192888.88[140643.1,290568.88] | 173611.34[119705.12,245470.25] |
|  | **mucosal thickness** | 61945.55[45826.44,97989.4] | 72838.45[43879.81,134403.39] | 75067.8[51130.34,106322.75] | 69849.45[49253.08,88424.53] |
|  | **villus perimeter (µm)** | 1022.48[845.92,1282.84] | 1180.39[996.14,1424.79] | 1034.52[876.92,1217.78] | 986.79[843.98,1099.07] |
|  | **Goblet cells** | 27[15.75,38.25] | 32[20.75,42.5] | 30.5[20.75,41.25] | 25[15.75,47] |
| **cecum** | **mucosal height (µm)** | 314.98[279.21,368.88] | 327.02[287.49,364.57] | 353.87[286.76,399.23] | 291.57[259.64,322.66] |
|  | **Goblet cells** | 19[12,32] | 22[13,36.25] | 21.5[10.75,35.5] | 16[12,24] |
| **colon** | **mucosal height (µm)** | 266.17[225.95,369.8] | 300.85[239.01,378.3] | 310.39[270.24,419.33] | 261.78[238.83,310.27] |
|  | **Goblet cells** | 19.5[14.75,28] | 20.5[13,26] | 25[18.75,35.5] | 20[14.75,27] |
