# Supplementary material for: Dietary supplementation with Bacillus velezensis and Pichia guilliermondii improves growth performance through intestinal morphology and functionality enhancement in weaning piglets
Source: PLoS One. 2025 Dec 4;20(12):e0332920. doi: 10.1371/journal.pone.0332920 (PMC12677519; doi:10.1371/journal.pone.0332920)
Supplement: S2 Table — (DOCX) [file pone.0332920.s002.docx]

**Supplementary Table S2: Median ± interquartile range of villi dimension features at t2**

|  |  | **t2** | | | |
| --- | --- | --- | --- | --- | --- |
|  |  | **ctr** | **pre** | **pre/pro** | **pro** |
| **duodenum** | **villus width (µm)** | 212.6[182.11,268.21] | 191.63[155.11,263.27] | 190.25[157.98,245.49] | 194.88[163.18,261.65] |
|  | **villus height (µm)** | 693.09[608.98,773.04] | 688.49[573.18,770.85] | 699.54[627.35,785.29] | 599.45[548.96,674.14] |
|  | **crypt height (µm)** | 420.89[366.47,472.73] | 426.78[350.21,493.29] | 403.41[353.95,483.37] | 380.15[335.18,442.51] |
|  | **villus area (µm2)** | 125290.23[94352.69,152987.8] | 112828.43[85482.43,156559.57] | 109905.66[87809.75,145422.98] | 101438.85[79894.66,136392.3] |
|  | **area II (µm2)** | 477538.7[364009.94,610614.87] | 393157.45[297493.5,592685.94] | 420039.67[324484.56,583127.79] | 371249.29[277682.29,525177.45] |
|  | **mucosal thickness** | 291117.15[222034.12,346067.42] | 297572.22[208100.66,363698.47] | 288293.48[230582.58,351713.09] | 228079.12[183163.99,287507.54] |
|  | **villus perimeter (µm)** | 1680.61[1520.33,1882.17] | 1670.84[1453.8,1870.05] | 1664.35[1522.34,1902.1] | 1500.09[1365.03,1686.9] |
|  | **Goblet cells** | 20[15,29.25] | 41[36,45] | 38[28.75,70] | 34[28,39] |
|  | **Cleaved-caspase-3 positive cells %** | 7.14[4.89,8.89] | 2.99[1.81,4] | 1.83[0.93,2.87] | 2.2[1.08,3.25] |
|  | **Ki-67 positive cells %** | 7.04[4.08,16.18] | 12.76[10.9,15.13] | 8.33[6.74,11.22] | 13.36[12.3,14.72] |
| **jejunum** | **villus width (µm)** | 145.63[114.22,193.85] | 154.6[124.24,204.96] | 155.06[134.63,196.35] | 157.25[136.28,193.82] |
|  | **villus height (µm)** | 519.29[436.37,597.71] | 543.6[480.28,643.84] | 552.79[485.12,631.31] | 483.5[407.58,571.34] |
|  | **crypt height (µm)** | 307.79[253.82,370.49] | 325.36[272.35,374.71] | 297.14[246.49,365.12] | 303.17[239.03,353.49] |
|  | **villus area (µm2)** | 70246.05[47504.37,87555.19] | 83235.17[59750.6,100620.23] | 76914.8[59961.83,94918.81] | 64765[50017.24,82778.33] |
|  | **area II (µm2)** | 241411.55[165646.34,319544.23] | 277489.07[210557,369381.41] | 266661.32[210446.85,367824.84] | 238602.62[178771.41,320573.57] |
|  | **mucosal thickness** | 164857.93[113060.68,208419.22] | 183427.83[129900.04,233005.35] | 162002.16[127593.01,234542.92] | 136933.67[105906.69,187756.76] |
|  | **villus perimeter (µm)** | 1294.98[1110.27,1476.64] | 1349.07[1216.51,1554.03] | 1352.41[1200.79,1520.62] | 1211.01[1036.92,1418.16] |
|  | **Goblet cells** | 19[13,30] | 29.5[25,35] | 17.5[12.75,23] | 26.5[22,31.25] |
|  | **Cleaved-caspase-3 positive cells %** | 8.07[5.56,9.82] | 1.4[0.71,2.21] | 1.86[0.97,2.57] | 2.15[0.93,3.15] |
|  | **Ki-67 positive cells %** | 8.49[6.55,13.58] | 11.11[9.42,12.99] | 7.69[2.78,10.61] | 9.37[8.34,12.65] |
| **ileum** | **villus width (µm)** | 183.89[146.23,216.05] | 175.89[129.62,231.23] | 169.89[134.65,223.76] | 185.95[155.58,223.4] |
|  | **villus height (µm)** | 429.59[389.35,503.5] | 402.12[346.28,474.56] | 452.82[402.02,495.43] | 447.64[396.08,504.61] |
|  | **crypt height (µm)** | 256.69[225.43,311.5] | 237.02[188.61,278.56] | 271.02[236.46,307.21] | 252.11[199.37,325.71] |
|  | **villus area (µm2)** | 66953.93[54616.37,80441.06] | 61843.93[45061.2,86779.49] | 66012.41[50088.76,83913.63] | 72341.99[56975.95,91955.82] |
|  | **area II (µm2)** | 252874.03[196852.33,319059.87] | 230647.28[159071.33,336020.35] | 242325.79[181735.55,324048.54] | 267861.4[211157.6,342813.89] |
|  | **mucosal thickness** | 108512.67[86438.39,158474.52] | 96374.85[68960.99,130559.04] | 125228.96[97619.68,150973.04] | 114250.01[80018.03,155970.98] |
|  | **villus perimeter (µm)** | 1150.16[1022.06,1303.78] | 1128.19[947,1269.48] | 1156.35[1055.34,1270.01] | 1188.68[1053.65,1374.78] |
|  | **Goblet cells** | 36[23.75,55] | 38[33,42.25] | 28[21.75,37.25] | 31[27,36.25] |
| **cecum** | **mucosal height (µm)** | 352.86[319.45,383.1] | 350.96[314.76,407.95] | 354.13[308.09,414.46] | 351.59[317.69,423.09] |
|  | **Goblet cells** | 14.5[9,22] | 36.5[26,43] | 22.5[15,30.25] | 35.5[29,40] |
| **colon** | **mucosal height (µm)** | 389.15[337.82,423.17] | 393.31[328.2,433.14] | 395.18[341.48,460.64] | 370.09[311.01,623.71] |
|  | **Goblet cells** | 22[15,29.25] | 41[35,48] | 25[17.75,35.5] | 39.5[35,43.25] |
