# Supplementary material for: Dietary supplementation with Bacillus velezensis and Pichia guilliermondii improves growth performance through intestinal morphology and functionality enhancement in weaning piglets
Source: PLoS One. 2025 Dec 4;20(12):e0332920. doi: 10.1371/journal.pone.0332920 (PMC12677519; doi:10.1371/journal.pone.0332920)
Supplement: S3 Table — (DOCX) [file pone.0332920.s003.docx]

**Supplementary Table S3: Median ± interquartile range of villi dimension features at t3**

|  |  | t3 | | | |
| --- | --- | --- | --- | --- | --- |
|  |  | **ctr** | **pre** | **pre/pro** | **pro** |
| **duodenum** | **villus width (µm)** | 234.21[192.12,277.13] | 232.81[188.2,290.51] | 247.27[193.5,290.13] | 215.7[173.42,286.37] |
|  | **villus height (µm)** | 711.37[603.24,840.79] | 760.29[681.17,830.09] | 762.56[689.15,859.44] | 728.2[615.01,800.62] |
|  | **crypt height (µm)** | 455.76[380.05,543.47] | 490.09[413.36,552.57] | 469.34[406.07,523.21] | 496.49[429.53,567] |
|  | **villus area (µm2)** | 143757.8[105417.7,184601.32] | 156078.04[123420.77,197680.25] | 158586.04[125005.22,188574.63] | 133916.83[104990.96,193999.38] |
|  | **area II (µm2)** | 520902.4[383048.68,710195.63] | 533147.4[433794.06,724906.65] | 602072.67[470977.43,706522.65] | 484191.48[367961.6,680629.72] |
|  | **mucosal thickness** | 317567.41[246220.01,441098.34] | 374246.74[288613.04,430047.92] | 345529.13[284767.25,428836.79] | 364336.58[272452.51,440232.07] |
|  | **villus perimeter (µm)** | 1772.43[1557.63,2044.22] | 1885.11[1690.06,2078.75] | 1876.25[1687.64,2081.7] | 1774.6[1599.73,1991.81] |
|  | **Goblet cells** | 40.5[36,46] | 38[34,42] | 40.5[35.5,45] | 37[34.75,41] |
| **jejunum** | **villus width (µm)** | 169.46[134.41,207.23] | 175.12[136.75,208.97] | 179.09[147.31,215.82] | 150.75[119.63,185.59] |
|  | **villus height (µm)** | 533.26[479.39,605.83] | 551.24[468.48,617.22] | 646.55[526.42,734.08] | 544.47[490.05,648.99] |
|  | **crypt height (µm)** | 317.98[281.46,367.07] | 348.56[292.22,415.47] | 359.83[312.8,414.43] | 355.38[303.14,420.04] |
|  | **villus area (µm2)** | 80483.78[64733.14,103136.37] | 80999.98[65368.05,108046.35] | 93126.41[74539.17,125470.32] | 78691.86[62015.63,98699.39] |
|  | **area II (µm2)** | 289237.55[217513.48,372662.15] | 290276.46[226173.39,387765.13] | 336749.71[265059.3,460987.09] | 259485.48[202866.31,351097.57] |
|  | **mucosal thickness** | 169978.46[134812.74,224051.77] | 182940.28[142836.05,262162.67] | 234720.5[173728.19,288523.36] | 195150.61[148632.97,265412.69] |
|  | **villus perimeter (µm)** | 1345.85[1206.39,1493.09] | 1364.65[1215.14,1535.47] | 1553.44[1307.11,1750.79] | 1370.24[1220.92,1563.23] |
|  | **Goblet cells** | 31[26,37] | 34[30.75,38] | 31[26,33] | 33[25.75,37.5] |
| **ileum** | **villus width (µm)** | 193.45[149.79,249.3] | 181.71[149.77,244.6] | 170[137.98,219.05] | 171.96[131.43,224.4] |
|  | **villus height (µm)** | 512.77[452.58,572.62] | 495.27[436,569.49] | 488.15[431.27,592.97] | 502.28[423.34,557.03] |
|  | **crypt height (µm)** | 287.13[243.12,342.78] | 278.32[243.29,344.93] | 296.07[244.25,364.32] | 279.1[231.9,328.19] |
|  | **villus area (µm2)** | 85282.95[63656.26,105353.86] | 86963.47[68022.08,113046.02] | 80272.3[58664.61,100159.34] | 75289.74[60117.6,92350.35] |
|  | **area II (µm2)** | 314992.21[233278.42,421432.67] | 303748.4[226284.86,397401.61] | 273562.06[220828.99,351930.52] | 267762.79[207921.7,337447.22] |
|  | **mucosal thickness** | 139870.95[114117.93,195517.2] | 136563.44[107759.92,192956.71] | 141580.77[109001.86,214832.8] | 133424.92[100200.3,182116.11] |
|  | **villus perimeter (µm)** | 1320.33[1176.52,1492.98] | 1319.97[1192.68,1490.63] | 1302.63[1136.23,1475.24] | 1282.37[1129.83,1424.45] |
|  | **Goblet cells** | 37[32.75,44] | 32[28,36] | 33[30,37] | 33.5[27,37] |
| **cecum** | **mucosal height (µm)** | 367.24[319.82,457.56] | 447.81[380.08,503.11] | 429.01[382.39,474.64] | 439.45[381.4,533.65] |
|  | **Goblet cells** | 39[35.75,42] | 34.5[32,43] | 39[35,42.25] | 34[27.75,41] |
| **colon** | **mucosal height (µm)** | 377.29[336.21,413.26] | 457.52[373.06,491.05] | 446.65[395.67,504.79] | 450.62[410.9,527.73] |
|  | **Goblet cells** | 42[39,48] | 43[38,48] | 45[41,51] | 37[33.75,43] |
