# Supplementary material for: Dietary supplementation with Bacillus velezensis and Pichia guilliermondii improves growth performance through intestinal morphology and functionality enhancement in weaning piglets
Source: PLoS One. 2025 Dec 4;20(12):e0332920. doi: 10.1371/journal.pone.0332920 (PMC12677519; doi:10.1371/journal.pone.0332920)
Supplement: S4 Table — (DOCX) [file pone.0332920.s004.docx]

**Supplementary Table S4 Linear regression analysis**

| Model Coefficients - weigth | | | | | | | | | | | |
| --- | --- | --- | --- | --- | --- | --- | --- | --- | --- | --- | --- |
| **Predictor** | | **Estimate** | | **SE** | | **t** | | **p** | | **Stand. Estimate** | |
| Intercept ᵃ |  | 3956.56324 |  | 8015.09963 |  | 0.49364 |  | 0.625 |  |  |  |
| duodenum villus width |  | 103.34759 |  | 43.65160 |  | 2.36756 |  | 0.024 |  | 0.95989 |  |
| duodenum villus height |  | 75.74970 |  | 43.55631 |  | 1.73912 |  | 0.091 |  | 1.87107 |  |
| duodenum crypt height |  | -12.22067 |  | 18.85444 |  | -0.64816 |  | 0.521 |  | -0.23732 |  |
| duodenum villus area I |  | 0.17806 |  | 0.09708 |  | 1.83416 |  | 0.075 |  | 1.66432 |  |
| duodenum area II |  | -0.06638 |  | 0.02890 |  | -2.29718 |  | 0.028 |  | -2.27584 |  |
| duodenum mucosal thickness |  | 0.00955 |  | 0.02462 |  | 0.38806 |  | 0.700 |  | 0.21647 |  |
| duodenum villus perimeter |  | -30.92987 |  | 20.23048 |  | -1.52887 |  | 0.136 |  | -1.84948 |  |
| jejunum villus width |  | 104.21438 |  | 53.08090 |  | 1.96331 |  | 0.058 |  | 0.62955 |  |
| jejunum villus height |  | 114.52268 |  | 45.19769 |  | 2.53382 |  | 0.016 |  | 2.43188 |  |
| jejunum crypt height |  | -9.35253 |  | 22.56655 |  | -0.41444 |  | 0.681 |  | -0.14664 |  |
| jejunum villus area I |  | 0.23343 |  | 0.12462 |  | 1.87310 |  | 0.070 |  | 1.14554 |  |
| jejunum area II |  | -0.07519 |  | 0.03774 |  | -1.99209 |  | 0.054 |  | -1.36933 |  |
| jejunum mucosal thickness |  | 0.01894 |  | 0.03292 |  | 0.57546 |  | 0.569 |  | 0.27022 |  |
| jejunum villus perimeter |  | -58.92456 |  | 22.85987 |  | -2.57764 |  | 0.014 |  | -2.85206 |  |
| ileum villus width |  | -48.01647 |  | 50.13876 |  | -0.95767 |  | 0.345 |  | -0.40517 |  |
| ileum villus height |  | -51.99286 |  | 47.25864 |  | -1.10018 |  | 0.279 |  | -0.85991 |  |
| ileum crypt height |  | 1.63065 |  | 17.21113 |  | 0.09474 |  | 0.925 |  | 0.02095 |  |
| ileum villus area I |  | -0.10697 |  | 0.09305 |  | -1.14954 |  | 0.258 |  | -0.53472 |  |
| ileum area II |  | 0.04318 |  | 0.03687 |  | 1.17130 |  | 0.250 |  | 0.74057 |  |
| ileum mucosal thickness |  | 0.01394 |  | 0.02733 |  | 0.51016 |  | 0.613 |  | 0.16817 |  |
| ileum villus perimeter |  | 17.24794 |  | 19.89581 |  | 0.86691 |  | 0.392 |  | 0.64641 |  |
| cecum mucosal height |  | 11.16703 |  | 4.48989 |  | 2.48715 |  | 0.018 |  | 0.17771 |  |
| colon mucosal height |  | -4.61992 |  | 3.17453 |  | -1.45531 |  | 0.155 |  | -0.09863 |  |
| duodenum Goblet cells containing sulfomucin |  | -8.07711 |  | 41.54153 |  | -0.19443 |  | 0.847 |  | -0.01568 |  |
| jejunum Goblet cells containing sulfomucin |  | -35.88692 |  | 55.18254 |  | -0.65033 |  | 0.520 |  | -0.04189 |  |
| ileum Goblet cells containing sulfomucin |  | 18.24845 |  | 36.02274 |  | 0.50658 |  | 0.616 |  | 0.02450 |  |
| cecum Goblet cells containing sulfomucin |  | -18.25310 |  | 36.61836 |  | -0.49847 |  | 0.621 |  | -0.03420 |  |
| colon Goblet cells containing sulfomucin |  | -2.50810 |  | 39.75874 |  | -0.06308 |  | 0.950 |  | -0.00535 |  |
| time: |  |  |  |  |  |  |  |  |  |  |  |
| t2 – t1 |  | 1354.39697 |  | 1550.61692 |  | 0.87346 |  | 0.389 |  | 0.26191 |  |
| t3 – t1 |  | 8590.50833 |  | 2031.43958 |  | 4.22878 |  | < .001 |  | 1.66118 |  |
| treatment: |  |  |  |  |  |  |  |  |  |  |  |
| pre – ctr |  | 732.60277 |  | 851.78743 |  | 0.86008 |  | 0.396 |  | 0.14167 |  |
| pre-pro – ctr |  | 2144.56612 |  | 695.76636 |  | 3.08231 |  | 0.004 |  | 0.41470 |  |
| pro – ctr |  | 894.19875 |  | 774.35007 |  | 1.15477 |  | 0.256 |  | 0.17292 |  |
| ᵃ Represents reference level | | | | | | | | | | | |
|  |  |  |  |  |  |  |  |  |  |  |  |
